# The influences of mindfulness on high-stakes mathematics test achievement of middle school students

**DOI:** 10.3389/fpsyg.2023.1061027

**Published:** 2023-04-06

**Authors:** Haode Zuo, Lidong Wang

**Affiliations:** ^1^College of Mathematical Science, Yangzhou University, Yangzhou, China; ^2^Collaborative Innovation Center of Assessment of Basic Education Quality, Beijing Normal University, Beijing, China

**Keywords:** mindfulness, meditation, high-stakes test, test anxiety, mathematics anxiety, mathematics test anxiety, mathematics self-efficacy, mathematics achievement

## Abstract

Research has shown that mindfulness can reduce students' negative emotions associated with high-stakes tests and thereby improve test performance. This study explored the association between mindfulness-based intervention (MBI) and high-risk math test scores of middle school students, which is noticeably slim in the domain of mathematics education, through a mediating process involving math-specific test anxiety and math self-efficacy. Using data from a sample of 45 students, age 12–13, we found empirical support for a significant positive correlation between mindfulness and middle school students' math achievement. Participants listened to a mindfulness audio every other week before a mathematics test. Weekly mathematics test scores, student group discussion data, and in-depth interview data were analyzed to explore how mindfulness affected students' mathematics test performance, which showed a statistically significant improvement after mindfulness compared to mathematics achievement without the intervention. Our results indicate that mindfulness can relieve mathematics anxiety symptoms, including physiological manifestations, test-unrelated thinking and worries, and problem-solving obstacles caused by mathematics anxiety. Also, mindfulness, especially its non-judgmental attitude, positively affects students' mathematical self-efficacy. The current research provides evidence of the mindfulness intervention's efficacy for improving middle school mathematics test performance but also identifies the complexities of implementing it with large numbers of students.

## 1. Introduction

Middle school mathematics tests are high stakes because they directly impact students, teachers, and schools (Reback et al., 2014). In many countries, mathematics academic performance is directly linked with students' graduation and continued study (Ho, 2009; Stobart and Eggen, 2012; Suprapto, 2016), which is especially significant in the test-oriented education environment of East-Asian countries. As a result, students in East-Asian countries are probably more anxious to do well in high-stakes mathematics tests (Tan and Yates, 2011). Current meta-analyses have shown that high-stakes tests are a kind of high-cognitive-demand competition, assessing students' cognitive competencies and ability to cope with anxiety in high-stakes testing situations (Burke, 2010; Keng et al., 2011). High-stakes mathematics tests often create a high anxiety situation for students, with mathematics anxiety playing a particularly large role (Bellinger et al., 2015). Empirical studies (Ashcraft and Moore, 2009) show that 17% of the population has high mathematics anxiety levels, and 33% of 15-year-old students in 65 countries who participated in the 2012 Program for International Student Assessment (PISA) reported feeling anxious when solving mathematics problems (OECD, 2013).

Moreover, improperly addressed mathematics anxiety will lead students into a vicious circle, where high-stakes mathematics tests cause mathematics anxiety, which causes further difficulties with mathematics (Maloney and Beilock, 2012). A comprehensive review found converging evidence of higher mathematics achievement usually accompanies a reduction in mathematics anxiety (Hembree, 1988). Further, a large number of empirical studies show that students with moderate mathematical self-efficacy often have a correct evaluation of their mathematics ability, but those with too high or too low mathematical self-efficacy will wrongly estimate their capacity, leading to excessive mathematics anxiety (Betz, 1978; Cooper and Robinson, 1991; Pajares and Urdan, 1996).

Recognizing middle school students' mathematics anxiety and improper sense of self-efficacy related to high-stakes tests, we posit that introducing mindfulness, a mode of attending to present moment experiences without judgment or elaboration (Bishop et al., 2006), may help. Previous work has suggested that mindfulness effectively improves academic performance by supporting students' ability to stay psychological well-being in the context of high cognitive demand (Napoli et al., 2005; Shapiro et al., 2011; Meiklejohn et al., 2012). Individuals tend to be more anxious in situations requiring high cognitive demand (Sarason, 1984; Schwarzer and Quast, 1985), especially high-stakes test situations. For example, when taking a high-stakes test, students' mathematics anxiety will give rise to test-irrelevant thoughts, e.g., about how hard the math test is or the consequences of failing the test. A growing body of evidence (Bellinger et al., 2015; de Abreu Costa et al., 2019) suggests that mindfulness provides a means to cope with and effectively buffer against the deleterious effects of anxiety. Additionally, robust research evidence indicates that mindfulness positively affects self-efficacy (Vidic and Cherup, 2019) and is the most significant determinant contributing to it (Chan et al., 2021).

Mathematics is assumed to elicit more substantial anxiety than most other academic subjects, such as literacy (Punaro and Reeve, 2012). However, there remains a lack of a microscopic examination of mindfulness's effects on improving mathematics performance in natural high-stakes testing environments. Thus, the present research investigated the impact of mindfulness within the context of a high-stakes mathematics test. Few studies have examined associations between mindfulness and students' high-stakes mathematics test scores, and even fewer have investigated how mindfulness-based intervention (MBI) affects students' performance. This study was the first to explore these two questions.

## 2. Theoretical background

### 2.1. High-stakes tests

Testing is considered high stakes when test results are referenced in important decisions affecting students, teachers, administrators, communities, schools, and districts (Haladyna et al., 1991). For example, high-stakes test scores affect students' graduation and promotion (Amrein and Berliner, 2002) and, in some cases, teachers' and principals' salaries and tenure (Council, 1998), as teachers and principals are often held accountable for students' high-stakes test performance (Jones et al., 2003). The effects of high-stakes tests on students differ across countries and cultures, but play a considerable role in East-Asian education, a test-oriented culture (Leung, 2001). For example, mainland China emphasizes that screening students' talents is an important national task. Historically, China's talent selection began during the Spring and Autumn Period, as self-recommendation. However, the country's destruction (despite many followers, such as the Four Gentlemen of the Warring States Period) showed this method was not ideal. The Han Dynasty initiated the approach of recommending people based on their filial piety and moral records, and the talented people selection system of Jin Dynasty revolves around nepotism and cronyism. Such methods had disadvantages, such as inconsistent reference standards, huge costs, and long talent training cycles. They also limited the generation of talents for the aristocracy, seriously aggravating social stratification and leading to reduced social mobility and other inequalities. The earliest high-stakes tests appeared in the imperial examination system established during the Sui and Tang Dynasties. For thousands of years, the advantages of high-stakes tests—such as low cost, high efficiency, high universality, and a high degree of standardization—were vividly demonstrated in the imperial examination and its evolution into various levels of entrance examinations. Of course, it had obvious drawbacks: high-stakes exams focus on measuring results, and such a strong sense of purpose goes hand in hand with a strong sense of crisis.

### 2.2. Math-specific test anxiety

Anxiety is an overarching construct, generally conceptualized as a state of emotion underpinned by qualities of fear and dread (Lewis, 1970). Mathematics anxiety has become increasingly prevalent in the past six decades, and its negative consequences for mathematics learning are well-documented (Dowker et al., 2016).

Mathematics anxiety, defined as a feeling of apprehension and fear related to mathematics (Ashcraft, 2002), is closely related to students' negative attitudes toward mathematics. First, mathematics anxiety comprises different components, often termed “cognitive” and “emotional.” Researchers have argued that mathematics anxiety's emotional dimension is more strongly negatively correlated with mathematics achievement than its cognitive dimension (Wigfield and Judith, 1988). Specific to high-stakes test situations, scholars argue that mathematics anxiety seems to be an aspect of “situational specific psychological distress” rather than an aspect of “mathematics” (Hembree, 1988). Neuropsychological data (Sheffield and Hunt, 2006) suggest that students' hearts may beat more quickly or firmly in high-stakes academic testing environments. Therefore, the relationship between mathematics anxiety and academic performance in a high-stakes testing environment needs careful consideration. Secondly, mathematics anxiety has various negative consequences, one of the most obvious being a decline or failure in academic achievement. A series of studies (Wu et al., 2012; Vukovic et al., 2013; Ching, 2017) has confirmed the negative link between mathematics anxiety and mathematics performance. Moreover, this negative correlation is particularly prominent among East-Asian students (Lin and Chen, 1995; Kirkpatrick and Zang, 2011).

Finally, Li (2001) found that individuals' thoughts when dealing with high-stakes mathematics exams commonly center on (a) the consequences of not performing satisfactorily and awkwardness when the actual situation does not meet their expectations, (b) feelings of inferiority and self-blame when they see other students' progress faster, and (c) clinging to familiar problem-solving methods. All these thoughts and behaviors, which are useless or harmful to solving test problems, can be concluded as thoughts unrelated to the current task and separated from the external environment (Stawarczyk et al., 2011).

Recent meta-analyses (Zhang et al., 2019; Barroso et al., 2021) provided findings to support that mathematics anxiety is largely in the form of math-specific test anxiety. Poor performance in a math test would lead to higher mathematics anxiety, whereas individuals who have higher levels of mathematics anxiety frequently get poor math performance. Overall, we believe the existing negative correlation between mathematics anxiety and mathematics performance is mainly talking in ways of math-specific test anxiety. In addition, as pointed out by empirical work (Bellinger et al., 2015), mindfulness might reduce specific manifestations of psychological problems raised by high-stakes testing situations. Thus, this paper explored mathematics anxiety in a high-stakes test context—math-specific test anxiety—which refers to abnormal mental experiences caused by excessive anxiety in mathematics test situations, especially high-risk mathematics test situations.

### 2.3. Mathematics self-efficacy

According to Bandura (2010), self-efficacy refers to the subjective conviction that one can successfully execute the behavior required to attain a desired outcome. He argued that people with high self-efficacy appraise their capabilities more favorably than those with low task self-efficacy, and this favorable self-appraisal leads them to outperform those with low self-efficacy.

One such kind of self-efficacy is mathematics self-efficacy, which refers to an individual's confidence in their ability to successfully perform or accomplish a specific mathematical task or problem (Betz and Hackett, 1983). Examples of mathematics self-efficacy include “I like to challenge mathematical problems,” “I hope mathematics can play an important role in my future work,” “When facing mathematical problems, I have the confidence and ability to solve them,” and “I am praised by my classmates and teachers because of mathematics,” etc. Studies support the theory that self-efficacy judgments are not mere reflections of past performance but thoughts about one's current situation or performance during the test (Schunk, 1982). Unsurprisingly, mathematics self-efficacy is negatively related to mathematics anxiety (Betz, 1978; Cooper and Robinson, 1991; Pajares and Urdan, 1996). First, a consistent finding of mathematics self-efficacy associated with mathematics anxiety is that individuals who experience mathematics anxiety express more negative attitudes about mathematics (Ashcraft, 2002), suggesting they lack confidence in their abilities. Further, Cassady and Johnson (2002) found that mathematics test anxiety contributes to increased worries and negative self-criticism, thus failing in high-stakes academic situations.

Unfortunately, prior research has taken a primarily static view of the relationship between mathematics anxiety and mathematics self-efficacy, without addressing the potential impact of change in external variables. Therefore, this study fills an existing research gap by exploring the interactive effects of mathematics self-efficacy and mathematics anxiety on students' mathematics achievement in high-stakes tests with the presence of the external variable, mindfulness.

### 2.4. Mindfulness

Beginning in the 1970s, international psychology and education scholars began to pay increased attention to mindfulness, leading to several definitions, such as “the awareness that emerges through paying attention on purpose, in the present moment, and non-judgmentally to the unfolding of experience moment by moment” (Kabat-Zinn, 2010, p. 145), and “a process of regulating attention in order to bring a quality of non-elaborative awareness to current experience and a quality of relating to one's experience within an orientation of curiosity, experiential openness, and acceptance” (Bishop et al., 2006, p. 234). The clinical psychology application of mindfulness has been altered and extended to many different fields through a variety of mindfulness courses, such as mindfulness workplace coaching (Ancona and Mendelson, 2014), childbirth and parenting (Duncan and Bardacke, 2010), competitive sports coaching (Scott-Hamilton et al., 2016), etc.

Of all possible applications, school-based mindfulness that combines students' psychosomatic characteristics and traditional school mental health curricula has received the most attention (Yu and Wenjie, 2017). School-based mindfulness has been widely used in recent years to help a growing number of students suffering from mental health problems, such as anxiety (Essau et al., 2011), depression (Jellinek and Snyder, 1998), and social withdrawal (Hipson and Coplan, 2017). Previous studies have confirmed the value of mindfulness applications for improving schoolwork. British and American scholars have found that mindfulness can alleviate students' depressive symptoms (Kuyken et al., 2013) and relieve stress (Wall, 2005) to a certain extent. Moreover, East-Asian scholars report that mindfulness improves students' concentration and performance in memory and other cognitive tasks (Ching et al., 2015; Lam, 2016). Likewise, scholars in mainland China have found that mindfulness training can improve middle school students' self-control (Changyu and Xiao, 2016), help relieve exam phobia, and prevent mental wandering (Shanshan and Zhun, 2018). As such, the common value of mindfulness applications across different fields is that they reduce the possibility of being disturbed by emotional factors in uncertain, painful, and anxious situations, such as job performance, pregnancy and childbirth, and high-intensity competition, thereby improving performance.

Most mindfulness therapies are categorized into two major branches based on their functional differences: mindfulness-based stress reduction (MBSR), which is used to help patients relieve anxiety, depression, and other emotional and psychological problems through meditation (Kabat-Zinn, 2003), and mindfulness-based cognitive therapy (MBCT). MBSR is a safe and effective treatment for reducing emotional deregulation, especially anxiety. Studies have found that MBSR outperformed in a group of individuals with a generalized anxiety disorder, suggesting it is superior in reducing anxiety symptoms (Evans et al., 2008; Vøllestad et al., 2011; Hoge et al., 2013). Recent studies have also examined the effects of MBSR intervention on positive states of mind and mindfulness self-efficacy, suggesting that mindfulness self-efficacy and positive states of mind are significantly higher after intervention (Chang et al., 2010). Similarly, MBCT has also been shown to effectively relieve anxiety (Chiesa and Serretti, 2011; Hofmann and Gòmez, 2017). Hence, mindfulness-based therapies are generally effective in reducing anxiety, stress, and depressive symptoms in adults (Baer, 2010) and children (Semple et al., 2010).

In addition, empirical studies and meta-analyses have found that mindfulness is the most significant determinant contributing to self-efficacy (Chan et al., 2021). In particular, Zeljka Vidic and colleagues found support for the impact of mindfulness on self-efficacy (Vidic and Cherup, 2019), indicating mindfulness can reduce test anxiety by improving students' sense of self-efficacy. Other findings suggest that a brief mindfulness intervention is an effective and practical means of enhancing academic self-efficacy and emotional well-being in university students, and a reliable and valid self-efficacy measure for mindfulness meditation practice has been developed (Birdee et al., 2020).

However, most school-based mindfulness studies rely on questionnaires (mainly self-reported student assessments) to assess the effects; few studies have measured the impact based on objective academic performance data. In particular, a common problem with questionnaires or self-report measures is that they may be affected by the accuracy and truthfulness of students' self-perceptions. Additionally, when students complete self-reports or questionnaires, they are not in an uncertain, painful, or anxious situation, limiting the research conclusions' validity. Therefore, studies are needed that obtain objective measures to assess the influence of mindfulness on student performance. Moreover, there has been little research on the efficacy of MBSR and MBCT interventions in high-stakes mathematics tests. We address this absence by using mindfulness interventions to ease students' math-specific test anxiety during high-stakes tests. Our research findings help explain how MBI enables students to get rid of math-specific test anxiety and enhance self-efficacy during testing, thus improving their academic performance.

### 2.5. The present study

As noted earlier, the current study's primary aim is to explore the process linking mindfulness to mathematics achievement in a high-stakes testing environment. Drawing on the extant literature (Franco et al., 2010; Bellinger et al., 2015; Samuel and Warner, 2021; Leppma and Darrah, 2022), we propose a theoretical framework that mindfulness interventions are beneficial for students' mathematics achievements in high-stakes exams situations by moderating the degree to which students were anxious about high-stakes mathematics tests and regulating mathematics self-efficacy to a normal level. The hypotheses for the present research study are:

1. Math-specific test anxiety will decrease in students receiving the mindfulness intervention.
2. Math self-efficacy (MSE) will increase in students receiving the mindfulness intervention.
3. The mindfulness intervention will improve students' high-stakes mathematics test achievements by reducing their math-specific test anxiety and increasing their math self-efficacy.

Two main research questions are addressed:

1. What is the relationship between mindfulness and students' high-stakes mathematics test achievements?
2. What are the underlying and intervening mechanisms in mindfulness and students' high-stakes mathematics test achievement relationships?

## 3. Research methods

### 3.1. Research participants

The stress resulting from exam crises is most apparent in middle school. At the primary school level, young students have not yet faced significant academic pressure; at the university stage, adult students have a relatively mature psychological bearing, examinations are typically not as intense, and there are opportunities to repeat exams after a poor performance. Therefore, the study was designed as a quasi-experiment, taking one natural class (45 students, ages 12–13 years, 30 boys and 15 girls) of eighth-graders in a junior middle school, conveniently selected from central Jiangsu province (China), as the subjects. As the sampled school is located in an urban area, participants largely identified as town dwellers. Consent was obtained from all participants, including their parents and relevant school personnel, before the study. The participants were similar to those students taking part in large-scale international comparative academic performance studies, such as The Program for International Student Assessment (PISA) and Trends in International Mathematics and Science Study (TIMSS). According to PISA 2018 (Schleicher, 2019), students in Jiangsu scored higher than the OECD average in mathematics. In addition, the overall mathematics level in the sampled class was relatively stable, and the scores of the recent two mathematics tests had not significantly fluctuated.

### 3.2. Research design

As students' academic performance followed (or approximately followed) a normal distribution, this study combined the characteristics of random variables subject to normal distribution. The G-power toolbox was used to calculate the required sample size to ensure sufficient statistical power. The class of 45 students was randomly chosen from a middle school, meeting G-power's sample size estimate (effect size = 0.5, 1 - α = 0.05, 1 - β = 0.95).

Applying the equivalent-time-sampling method shown in Table 1, quantitative survey and objective data were used to test research hypotheses. The experiment lasted 4 weeks and was divided into the experimental period (*X*) and control period (*X*_0_), each lasting 2 weeks. Data were collected weekly (i.e., four math test scores in 4 weeks (*O*_1_, *O*_2_, *O*_3_, *O*_4_). Students were not given mindfulness (*X*_0_) before the exam in the first and third weeks (i.e., the students were not guided to any mindfulness practice); in the second and fourth weeks, the same students received the intervention (*X*) before the exam (i.e., the students were guided to practice mindfulness by listening to a recorded audio of mindfulness).

**Table 1:** Equivalent-time-sample design.

**First cycle**	**Second cycle**
Math scores without mindfulness *X*_0_*O*_1_	Math scores without mindfulness *X*_0_*O*_3_
Math scores with mindfulness *XO*_2_	Math scores with mindfulness *XO*_4_

### 3.3. Research instruments

#### 3.3.1. Mindfulness-based intervention

The intervention involved students sitting in the classroom as a group before the test, guided by audio mindfulness. None of the many ready-made versions of mindfulness audio is custom-made for a math test. The researchers re-contextualized the concept, putting it into the framework of student psychological problems that may occur during a stressful high-stakes math examination. As discussed above, as MBSR and MBCT are commonly-used means of MBI, their operational definitions (Williams et al., 2007) were highlighted to ensure the essential connotations of mindfulness—intentional focus, non-judgemental attitude, and the present moment—were not lost. The first author acted as the MBI instructor. Having completed an online mindfulness training practicum hosted by Dr. Huiqi Tong, a teaching partner and chief Chinese translator of Kabat-Zinn, founder of the “MBSR” program, he possessed a basic level of training and sufficient proficiency to deliver MBI to the students. The researchers' audio recordings lasted 15 min; evidence (Zeidan et al., 2014) has shown that short-term mindfulness intervention is also effective for subjects without prior meditation experience. The main content of MBI in this current study is summarized below: At the beginning of the session, the instructor asked students to sit in a graceful, comfortable, and conscious posture, with their bodies straight, eyes slightly closed, shoulders down, and hands relaxed. The instructor then guided the students to breathe naturally, pay attention to their breath (without consciously controlling or adjusting it), be aware of the feeling it brought to their body's organs, focus on keeping their inhalation and exhalation natural, and gradually calm their heart. When students found themselves involuntarily distracted from their breathing, the instructor would tell them: “*Don't panic; you should know that distraction or mind-wandering is a natural occurrence, be aware of where it is, let go, and bring your mind back to your breathing.”*

Then, the instructor directed students to turn their attention to the body and feel the sensation of breathing on all body parts. If the student felt tense or uncomfortable during this process, the instructor would tell the student, “*Make sure they're there, don't try to change them, just put them down and get your attention back on your breath.”*

Next, the instructor led the students through an awareness exercise. At the beginning of the exercise, the instructor told the students: “*If you find yourself distracted, don't panic. That's normal. We can use the third person's perspective to see where our attention is going and whether it is becoming your thought. Just like our awareness of breathing, we only need to observe the emergence, change, and disappearance of thoughts. Now imagine that we are sitting in an exam room taking a math test. During the test, I would calculate how many points I got while being afraid of how many points I would lose if I made a mistake in this question. Then I began to think about how much my ranking would drop if I lost these points and what would happen after that. We all know it's unnecessary, but we can't get rid of it. Now, I'd like you to look at this thought and name it. Is it a plan, a recollection, a worry, or a fanciful image? You would find that it's a ‘worry', it's our habitual way of thinking, and it's not real. After naming, the thought usually loosens, disintegrates, and disappears. Notice the naming and the disappearance of the thought. Then, bring your attention back to your breath, to the present moment.”*

Through this process, the instructor used mindfulness to intervene in students' compulsive checking and being swayed by considerations of gain and loss, commonly seen when students participate in high-stakes tests. This achieved the “contextualization of mindfulness in high-stakes mathematics test situations” by using mindfulness's “intentional attention” to keep students focused on the test itself and the “non-judgemental attitude” and “present moment” in mathematics self-efficacy to tell students that worries are only thoughts, not facts, thus getting rid of expectations about how well they will do on the activity: “*For another example, when I took the test, I found that the questions were far from what I expected; it's different than usual. I began to scratch my head and lose my mind. At this point, we should also look at the thought, classify it, and discover that it's a plan, that it's not a fact, that I should turn my attention back to my breath, back to the present.”*

Through this process, the instructor used mindfulness to intervene in students' common problem of fixating on difficult questions, leading to a complete loss of confidence. He used mindfulness' “intentional attention” and “present moment” to tell students that “plans” are only thoughts, not facts, and ultimately improve their sense of self-efficacy, making “re-contextualized” mindfulness easier for students to accept, allowing them to participate in the intervention process actively.

Throughout the practice, the instructor guided students to observe their thoughts in a non-judgmental manner, telling them that instead of thinking about the exam results or expecting a challenging exam, it would be better to focus on the present moment without presupposing the results to enhance self-efficacy. Whether the thought itself or the emotion it caused were pleasant or unpleasant or there were feelings attached, there was no need to dwell on the content, just watch one thought after another as they rose and disappeared, and then continue.

Finally, the instructor led the students through an observing emotions exercise. Students were asked to take three deep breaths, watching for and patiently observing the emergence of any emotions as they breathed. Sometimes, several emotions would appear simultaneously; other times, there were no obvious feelings. The instructor told the students: “*Recognize and confirm any emotion, don't judge it but just experience the feelings it brings to your body; it's just a mental phenomenon and doesn't represent the truth. Don't try to change it. Similarly, imagine sitting in an exam room and taking a math test. During the test, I saw that other students solved problems quickly and reached the next page, but I still stayed on the previous page. I began to feel inferior and self-blamed, blindly following their speed. Now, let's look at this emotion and give it a name. Is it fear, insecurity, or loneliness? We find that it's insecurity. It's just a phenomenon of our mind and is not the same as a fact—we're not the same as the emotion. After naming, the emotion usually changes and goes away. Notice this process, and then bring the attention back to the breath and back to the present.”*

Throughout this process, the instructor used mindfulness to intervene in students' common problems of self-abasement and self-reproach, using mindfulness's “intentional attention,” “non-judgemental attitude,” and “present moment” to tell students that insecurity is only an emotion, not a fact. After being named, the emotion would change and disappear.

At the end of the intervention, the instructor prompted students to bring their minds back to reality and try to bring a relaxed, secure, and comfortable state of mindfulness into the upcoming exam.

#### 3.3.2. Math test

Most studies rely on questionnaire measures to assess for effects (mainly student self-report) and do not include follow-up assessments, which remains a limitation in the existing literature (Liehr and Diaz, 2010; Lau and Hue, 2011; Lagor et al., 2013; Metz et al., 2013; Felver et al., 2016). This study obtained student achievement and grades as objective data on student educational or behavioral outcomes. The experimental class's math teacher was responsible for making the test, compiling the questions, correcting the completed tests, and compiling the grades. The test consisted of free-response questions and was graded on a 10-point scale, the scoring rubric was inter-rater checked by the first author and the class's math teacher. Tests are ebbed in the participants' weekly quizzes, which are linked with their unit assessment, and may further imply their graduation assessment. And the tests are taken in the classroom, where the participants could receive peer pressure during the test-taking (Beilock et al., 2004; DeCaro et al., 2010). The math test used in this study was based on students' actual learning progress and chosen or adapted from the textbook or related exercises to ensure content validity. An analysis of student responses to each question showed a Cronbach's alpha of 0.864, indicating high internal consistency reliability and suggesting the math test had qualified and sufficient homogeneity reliability (Table 2).

**Table 2:** Reliability analysis.

**Cronbach's alpha**	**Numbers**
0.864	8

To ensure that the math tests used across the 4-week experiment were parallel, the difficulty and differentiation of four sets of questions were analyzed, and their difficulty was calculated using an extreme grouping method that ranked the weekly math scores in descending order. Starting from the highest score, 27% of the total volume was sampled as the high score; starting from the lowest score, 27% of the total volume was sampled as the low score. *P*_*H*_ and *P*_*L*_ represent the difficulty value of the test for high and low group candidates, respectively, and the difficulty value of the test was expressed by $P=\frac{P_{H}+P_{L}}{2}$. The results are shown in Table 3. The difficulty of the four groups of questions was between 0.6 and 0.8. *D* = *P*_*H*_ − *P*_*L*_ was used to represent the tests' degree of distinction. The results are shown in Table 3; the four groups' discrimination degree was above 0.4, indicating that the difficulty and differentiation of the four groups of questions were consistent. Therefore, the mathematical tests adopted in this 4-week experiment were equivalent in terms of homogeneity, equal reliability, fairness, etc.

**Table 3:** Difficulty and discrimination value.

**Week**	**Difficulty**	**Discrimination**
1	0.671	0.584
2	0.723	0.520
3	0.627	0.604
4	0.686	0.555

#### 3.3.3. Group discussion and in-depth interviews

At the end of the 4-week experiment, researchers interviewed students to understand whether they perceived the mindfulness intervention as helpful and, if so, why. A class meeting was held, attended by the 45 students. Students were given the math papers they had marked and were asked to review the mindfulness intervention's whole process and confirm their answers. Then they were asked to discuss “whether the mindful intervention had been helpful in the test” based on their test situation in the first 4 weeks. The students were divided into four groups, with one researcher chairing each group's discussion. The discussion process was recorded and included the following questions:

1. What psychological problems have been alleviated and improved after mindfulness practice?
2. What do you think the mindfulness practice did to your ability to successfully perform or accomplish a specific mathematical task or problem during the examination?
3. When you need to deal with these psychological problems again in a future exam, will you have a better way and more choices to deal with it?
4. Do you think the mindfulness practice was useless for you? If so, explain why.
5. What aspects could be improved in implementing mindfulness to make it easier for you to accept?

Each group was asked the questions in sequence; all participating students expressed their opinions.

After the group discussions, to better explore the impact and limitations of mindfulness, the researchers chose four students—two who had significantly improved and two who had not—to complete an in-depth interview on “what psychological factors mindfulness can influence regarding middle school students' math test scores.” Interviewee characteristics are shown in Table 4.

**Table 4:** Interviewee characteristics.

**Students**	**Gender**	**Scores**				**Improvement rate in Week 4 compared to Week 1**
		**Week 1**	**Week 2**	**Week 3**	**Week 4**	
*A*_1_	Male	9	10	9	10	11.11%
*B*_1_	Male	5	10	7	7.5	50%
*A*_2_	Female	9	10	9	10	11.11%
*B*_2_	Male	5	6	9	9	80%

Before the formal interview, the students were introduced to the study's purpose and promised their information would be kept confidential. Then the students were guided to review the mindfulness process and the math exam. One researcher asked questions based on the interview outline. For those who believed “mindfulness is useful to me” the main questions were about mindfulness' influence on their psychological problems under the stressful conditions of a high-stakes mathematics test and their achievement motivation during the exam. For those who believed “mindfulness is almost useless to me,” the main questions concerned why they could not integrate into the mindfulness environment. The other researchers were responsible for recording students' responses.

Interviews were transcribed verbatim with no analysis cuts through three processes: extraction, coding, and classification. The second author checked the transcript for accuracy, and the researchers manually coded it. We used a two-step coding system to capture the themes and codes to describe the mechanisms transmitting mindfulness' effects on students' mathematics achievements in high-stakes testing environments, first converting informal language into academic language (i.e., researchers first read through the transcript to grasp the answers, inductively deriving codes), then coding high-frequency key nouns (i.e., “relieve,” “enhance,” and “promote”). Constant comparison coding was conducted in which the researchers read the texts and highlighted related information about the underlying relationships among mindfulness, math-specific test anxiety, and mathematics self-efficacy, based on the literature and the researchers' understandings of mindfulness' effects in school contexts. Next, thematic categories were constructed based on the connections identified in the open coding process and aligned with the implemented theoretical framework. The first author and two research assistants conducted the coding process independently, resolving any coding disagreements through discussion (Cohen et al., 2017). The resulting coding themes and categories are presented in Table 5.

**Table 5:** Coding themes and categories.

**Math-specific test anxiety (MTA)**	**Mathematics self-efficacy (MSE)**
**Physiological manifestations:**	**Past experiences dictate students' opinions**
(a) Heart rate	**concerning their personal ability in mathematics:**
(b) Tight body	(a) Comparing self-performance to peers
	(b) Overconfidence or under-confidence in test performance
**Test-irrelevant thinking and worry:**	
**(**a) Excessive consideration of the	**Mathematics self-efficacy-related problem-solving obstacles:**
consequences of failure in this math exam	(a) Comparing problem-solving speed to peers, breaking own
(b) Sorrow for their parents	time arrangement for answering the test paper
(c) Undue frustration by running out of the exam time	(b) Excessive pursuit of ingenious problem-solving methods
	(c) Habitually walk away from any challenging
	mathematics problem
**Mathematics test anxiety-related problem-solving obstacles:**	
(a) Clinging on to some familiar problem-solving method	
(b) Obsessing on some unsolved problems, unable to	
adjust mind in time to solve the next problem	
(c) Excessive upset when the real difficulty of the test is	
inconsistent with the anticipated difficulty	

## 4. Results

This study employed mixed methods, with quantitative data as the main material and qualitative data as auxiliary material, to explore the mechanism of mindfulness' influence on students' high-stakes mathematics test performance, based on the same-time-sampling design applied to a middle school sample.

### 4.1. Changes in students' high-stakes test scores before and after mindfulness practice

**Figure 1:**
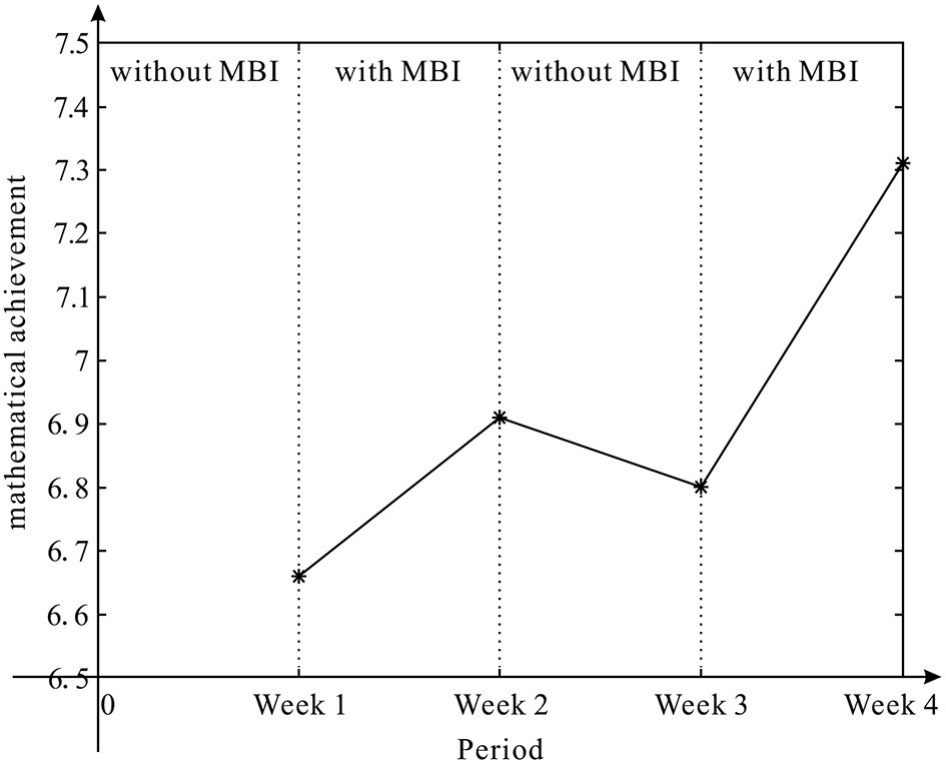
The average score of students in math test.

The average scores of all students from week 1 to week 4 were 6.66, 6.91, 6.80, and 7.31, respectively. The results are depicted graphically in Figure 1, where there are clear trends of increasing after receiving mindfulness intervention and a trend of decreasing after removing the intervention.

A one-sided *Z*-test was conducted on math score means from parallel tests in weeks 1 and 4 to examine whether mindfulness intervention could account for students' improvement in scores of tests. The results are shown in Table 6.

**Table 6:** Mean *Z*-test for math scores in weeks 1 and 4 (α = 0.05).

**Mean**		**Mean**		**95%CI**	
**Week 1**	**Week 4**	**Week 1**	**Week 4**	***p***	**Cohen's *d***
6.66	7.31	[5.95,7.36]	[6.60,8.02]	0.035	0.27

Differences were deemed significant when confidence limits exceeded 95% (*p* < 0.05). The results showed that the scores of d that followed mindfulness significantly improved compared to the tests' scores without the mindfulness intervention.

### 4.2. What mindfulness brings for math-specific test anxiety

Based on thematic analysis and synthesis of the qualitative data collected from group discussion and in-depth interviews, three broad themes were identified regarding mindfulness's influence on students' mathematics test anxiety in the high-stakes testing environment.

#### 4.2.1. Reduces physiological manifestations of MTA

The participants believed the mindfulness practice lowered their heart rate, mainly the guided breath frequency and body scan in the audio. In the beginning, the audio guided participants to breathe based on its rhythms (“*Close your eyes and take some deep, slow breaths in and out; with every breath in, relax your body a little bit more”*), and then asked them to “*feel the surface below you, relax your face, your tongue, your neck and your arms and your fingers...while breathing out relax your back, your chest, your hips, your legs, and your feet”*. The MBI taught students to pay attention to their bodies, making them feel their heart rate slow and assume a more relaxing posture before the exam, as some students stated in the group discussion:

“*I think it's helpful when I listen to the audio, like, you know, it makes me feel comfortable and lowers my heart rate. And the rhythm of my breath seems to be enough to change my brain, a move from anxious, more toward wellness...”*

“*...the moment in which during the body scan, I have a better awareness of my body organs. I have a better sense of my head, my neck, my shoulders, my chest... I can feel my arms by my side, feel my feet on the ground. Sometimes, I kept my shoulders shrugging when I was doing some homework. Listening to the audio, I feel my body's stretched, I can feel my arms by my side, feel my feet on the ground. In sum, I am able to find a comfortable position, I can find my body awareness, the sensory awareness in my body, I relax myself from head to foot, and it makes me feel at ease, relaxed, less anxious. I really enjoy this feeling.”*

#### 4.2.2. Reduces test-irrelevant thinking and worry

Aside from reducing the physiological manifestations of MTA, students shared that the MBI also reduced test-irrelevant thinking and worry. Analyzing the qualitative data describes three aspects of this effect:

- Reducing excessive consideration of the consequences of failure in these math exams
- Relieving sorrow for their parents
- Reducing undue frustration by running out of the exam time.

First, during the exam, students were obsessive about attaining a correct outcome and the consequences of failing the test. The mindfulness audio guided them to reduce MTA by refocusing on the pencil in their hand rather than worrying and obsessing about outcomes and consequences. The participants mentioned how test-irrelevant thinking and worrying might be reduced by the MBI:

“*I often go down the alarming path of thinking. I just cannot get rid of ‘what happens if?' What happens if I have miscalculated? What happens if my teacher penalizes me for a proof mistake or a misplaced symbol? The audio describes it as that our lives are continuous exceptions of ups and downs, twists and turns of agreeable and disagreeable situations. I noticed that lives are based on the present moment. So my focus should be on making the present moment the best we can. I gradually free myself from these disturbing and worrying thoughts, I find a kind of concentration arise, and I am not that anxious.”*

“*Every time I hear my mother's relatives say some other children are good at math and look proud, I wonder when I can make my parents so happy. And every time, it seems the more I want to satisfy my parents by performing well on a math test, the more disappointed my parents may get. I already feel dumb instead of anxious about this... however, after receiving the MBI, I realize that being in the past ruminating or regretting events is not helpful at all. Hence, I set aside thoughts of sorrow for my parents, and I pay attention to the test moment in an open, accepting way to what I am experiencing.”*

“*I am kind of a slowpoke person. When I was in grade one or two, it was not until ten in the evening before I could finish writing my homework. After entering junior high school, it is increasingly common for me to be unable to complete the math paper in regular time. This situation almost made me in a vicious circle. The more frustrated I am by running the exam time, the less I can concentrate on problem-solving. With the help of the MBI, instead of frustrating for not having enough time, I started to spend longer concentrating on the present problem-solving and became more focused and engaged. Finally, to my surprise, I did finish the exam paper before the bell rang. It comes to me that maybe because if I always think about the due time, my focus is constantly on the future, and not on the work that needs to be done at that very moment.”*

#### 4.2.3. Reduces MTA-related problem-solving obstacles

MBI can also be seen as essential in helping students surmount mathematics test anxiety-related problem-solving obstacles. Participants said they thought that MBI could stop them from:

1. clinging to familiar problem-solving methods;
2. obsessing over unsolved problems and help them adjust their minds in time to solve the next problem;
3. being excessively upset when the test is more difficult than anticipated.

*B*_1_ and *B*_2_ all particularly mentioned that these mindfulness exercises are helpful in mathematics problem-solving:

- *B*_1_: “*In solving the problem ‘Given the height and distance of two straight rods, the distance between the top of the rod and a certain point is equal, find the horizontal distance between the bottom of the rod and that point,' I no longer had the thought of clinging on to some familiar problem-solving methods, for example, proving the congruence of two triangles. I told myself that I should focus my attention on the present problem, not on the previous problem or not on congruent triangles on seeing line segments equal. And then, when I got down to it, I realized that I could solve this with the Pythagorean Theorem... ‘Given the perpendicular bisector of the hypotenuse of a right triangle, find the distance from its intersection to the vertex of the right angle'; this problem, which we rarely encountered in our daily study, belongs to the new type of question. Faced with this new type of question, I was no longer confused, no longer subconsciously comparing it with the old type of question and judging how much I could score. Instead, I broke my expectation about score and began to consciously focus my attention on the question itself and look for solving strategies. Finally, I got the answer by adding auxiliary lines and combining the Pythagorean Theorem and vertical bisectors' properties.”*
- *B*_2_: “*In solving the problem ‘Given the height and distance of two straight rods, the distance between the top of the rod and a certain point is equal, find the horizontal distance between the bottom of the rod and that point,' I found that I could not prove the congruence of triangles with the methods I had mastered, nor did I know of any other methods. Also, I found myself spending too much time on this problem, so I decided to take the plunge. I guessed the answer according to the existing numbers in the question, and I told myself that the answer which I think is correct is probably the right one. And it would be difficult for the teacher to be random when setting the question. Instead of trying to solve this question, I would rather pay attention to the details in the question stem and guess the answer first. Finally, my guess turned out to be the right answer.”...“I didn't know where to start when I answered the question ‘draw a specified square and triangle in a square grid,' and then I chose to do the next one first. At this point, I will just focus on the present problem and not think about the previous problem. When I finish this problem, I will use the rest of the time to think back to the previous problem.”*

### 4.3. What mindfulness brings for math self-efficacy

Based on the analytic framework (Table 2), two general themes emerged from our group discussion and in-depth interviews regarding mindfulness' effects on participants' MSE, as identified below.

#### 4.3.1. Gain a better understanding of the judgment and evaluation of MSE

According to the participants, an non-judgemental attitude—an essential element of mindfulness practice—enabled them to recognize and understand their actual MSE concerning their experience. Two themes came to light in terms of the MBI's perceived effects:

1. students stopped comparing their performance to their peers', and,
2. students reduced their overconfidence and underconfidence regarding math test performance.

Students suggested, in the group discussion, how MBI might help them maintain a more general and practical understanding of their MSE:

“*Part of my mind is always comparing. If I compare myself to people better than me, I gonna be unhappy... Sometimes, I use harsh self-criticism, based on my results of the comparison with peer students, to motivate myself to study... The awareness and the wisdom I gain from mindfulness work together, helping to reduce the time spent in judgment and evaluation of the past experiences dictate my opinions concerning my ability in mathematics.”*

“*I believe myself to be better than others in mathematics. Therefore, I may sometimes be overconfident in a math test, and it is more likely for me to make some stupid mistakes in the exams. This kind of mathematics self-efficacy sometimes annoys me. I have been punished for my carelessness in both math tests and math homework. My carelessness used to cost me first place on the test several times… through mindfulness, I have an awareness of knowing what my mind is doing. I slow down and read the questions fully, and I create an outline for the geometrical proof, which is good for the organization before I start writing. This may help me to avoid making stupid mistakes or random mistakes on exams.”*

“*For me, studying mathematics is a negative experience. I used to think that I was not a math person. I used to have a weak will in exams because of my slow thinking. When I found that people were all the better than me in mathematics, I would become more cynical and have low exam results expectations...At every moment, and although I know that I do not have any control over what has happened in the past. Instead of comparing myself to others, feeling guilt, resentment, bitterness, and sadness on my previous mathematics experience, I shall constantly focus on what I will achieve in this present math test.”*

#### 4.3.2. Reduces MSE-related problem-solving obstacles

The following three themes emerged from our qualitative research regarding how students' judgments about their ability to accomplish the math test (mathematics self-efficacy-related) affected the obstacles to their mathematics problem-solving. Specifically, students:

1. compared their problem-solving speed to their peers', breaking their time arrangements for answering the test paper;
2. excessively pursued ingenious problem-solving methods; and,
3. habitually walked away from challenging mathematics problems.

Respondents indicated that MBI could help them through the above difficulties:

“*I'm a little slow at how to find if triangles are congruent, and in the past, I'd start noticing if everyone around me had already constructed congruent triangles, and then I'd blame myself for not being able to solve them. After taking part in the mindfulness intervention, I stopped judging my and others' math ability and focused my attention on the present through the senses. I no longer pay attention to the speed of the people around me, no longer compare myself to others, and question and deny my ability based on others' progress. Instead, I only pay attention to the problem and identify the appropriate one from the five rules to prove triangles congruent.”*

“*I think I've always been good at math. Therefore, I was accustomed to comparing and analyzing various solutions before starting to solve a test question. For example, when facing the item ‘draw a triangle with sides 3*,$\sqrt{10}$,$\sqrt{13}$ *of in the square grid (Week 3 test),' though some conventional methods could solve it, I just want to find a different triangle that could make my solution remarkable. And it turns out not gratifying because this wastes me a lot of time. To be honest, I sometimes pursue more skilful solutions, which could distinguish me from other students. Somehow, it is tough to find an ingenious solution in the examination situation, and it seems to be the loss outweighs the gain. Mindfulness told me that I should find my inner power and constructively work with what I think is the best at each given moment instead of constantly striving toward the new and better based on my judgment.”*

“*I tend to put more weight on my past failures when challenging the challenging question in the test. This negative bias keeps me habitually running away from those difficult problems, usually the second question of the item. Then mindfulness told me that the outcome of any particular situation is not determined by the factors we went into. If we change the factors, we can continuously change the results. Instead of judging events that haven't happened yet, and, frankly, may never happen, we should be open and accepting. Hence, I have a good attitude toward those challenging items and challenged the last item's second question on the Week 4 test. Though I didn't get it totally done, I still get some points on this item.”*

## 5. Discussion and conclusion

This study has examined the relationship between mindfulness and middle school students' high-stakes mathematics test achievement by exploring the association between mindfulness, MTA, and mathematics self-efficacy. The *p*-value and effect size values revealed significant positive correlations between mindfulness and middle school students' mathematics achievement. Semi-structured interview results provided evidence of the mediational mechanism between mindfulness and MTA. The relationship between mindfulness and mathematics achievement was partially mediated through MTA and mathematics self-efficacy. Our results are somewhat similar to those reported by previous studies, though these are noticeably slim in the domain of mathematics education.

First, the findings support previous research indicating positive associations between mindfulness and academic achievements (Beauchemin et al., 2008; Lu et al., 2017; Miralles-Armenteros et al., 2021). In particular, Bellinger et al. (2015), the first to investigate the connection between mindfulness and mathematics performance in high-stakes academic testing environments, found that mindfulness indirectly benefited math performance by attenuating the harmful effects of test anxiety. Also, consistent with Franco et al. (2010) and Samuel and Warner (2021), the relationship between mindfulness and academic performance in this study was mediated by anxiety and self-efficacy. Although there is no quantitative support for this mediating mechanism, our research has highlighted robust qualitative supporting evidence. More specifically, mindfulness appears to clear up symptoms of MTA disorders, including physiological manifestations, test-irrelevant thinking and worry, and mathematics test anxiety-related problem-solving obstacles. Also, mindfulness, especially adopting a non-judgmental attitude, moderated students' mathematics self-efficacy. Mindfulness can make students more cognizant of and confident in their ability to perform or accomplish specific mathematical tasks or problems; in other words, students with a non-judgmental attitude can think of their abilities better and stay focused on the “present-moment” in high-stakes tests, thereby better accessing their mathematics self-efficacy. Table 7 may largely subsume participants' ideas according to the zone patterns of the “past,” the “present,” and the “future,” which may reveal the underlying and intervening mechanisms in mindfulness and students' high-stakes mathematics test achievement relationships.

**Table 7:** The classification of participants' ideas according to the zone patterns of the “past,” the “present,” and the “future.”

**Zone patterns**		
**Past**	**Present**	**Future**
1. Sorrow for their parents. 2. Comparing self-performance to peers. 3. Clinging on to some familiar problem-solving method. 4. Excessive upset when the real difficulty of the test is inconsistent with the anticipated difficulty. 5. Habitually walk away from any challenging mathematics problem. 6. Obsessing on some unsolved problems, unable to adjust mind in time to solve the next problem.	Paying attention in the moment, in an open and accepting way, to what you are experience in high stakes math test.	1. Excessive consideration of the consequences of failure in this math exam. 2. Excessive pursuit of ingenious problem-solving methods. 3. Comparing problem-solving speed to peers, breaking own time arrangement for answering the test paper.

Second, the current research does not explore how working memory may apply to understanding the phenomenon of mathematics anxiety as reported in previous studies (Mandler and Sarason, 1952; Eysenck and Calvo, 1992; Ashcraft and Moore, 2009; Berggren and Derakshan, 2013). Many studies have found it (Hitch, 1978; Gathercole and Pickering, 2000; Swanson and Sachse-Lee, 2001) over the years, indicating that the deficit of working memory would have a powerful effect on arithmetic. One reason could be that the math test used in this study was on geometry rather than arithmetic, which requires a more robust understanding of geometrical theorems (i.e., the Pythagorean Theorem, the Condition of Triangle Congruence, and the Property of Congruent Figures). This, in turn, requires less working memory and might interfere less with the mathematics anxiety associated with working memory deficits.

Third, in-depth interviews with students who did not show significant improvement also showed they viewed mindfulness as “mysterious” and “magical.” They believed mindfulness was equivalent to Buddhism and could not fully devote themselves to the intervention during the experiment; “The whole audio gave me a sense of mystery. As a result, I don't feel fully immersed in the environment that the audio provides.” Therefore, mindfulness needs to be demystified and re-contextualized for the educational context so students can more easily understand and accept the intervention. “Demystifying” involves integrating the Buddhist dharma of mindfulness into students' daily school life in a language that maintains its original meaning without mentioning the term “Buddha dharma.” “Re-contextualization in the educational context” refers to comprehending mindfulness in an educational context. The connotation of mindfulness is explained based on the difference between Buddhist and educational contexts. Through attentional intervention, students are taught to be aware of their “bad” psychology and behaviors in their daily studies and examinations, treat them with a non-judgemental attitude, accept them, and stay in the present moment.

Furthermore, mindfulness showed effects related to religious concepts on students' exams:

“*During the exam, I would tell myself that the result is a foregone conclusion. What I need to do at this moment is to try my best to do well in the questions that I know the answer. As long as I did my best in the exam, the score is not what I need to worry about. After I had got involved in the intervention, I found that my expectation for the score would not disturb my rhythm anymore.”*

Although researchers emphasized demystifying mindfulness and re-contextualizing it in an educational context, mindfulness cannot eliminate the Buddhist context in some students' minds. Accordingly, identifying ways to handle this aspect should be a priority in future research.

As with all research, the current research has both strengths and limitations. From a methodological viewpoint, a major benefit of our study is its mindfulness instrument. The mindfulness audio in this study was more tailored for a mathematics test than those used in previous studies in that it targeted students' math test performance in an actual high-stakes test situation. However, the study's methodological limitations should be considered when interpreting its findings, particularly its lack of a randomized design; participants were limited to a convenient sample drawn from a single school, which limits generalizability. Future research should employ more extensive and diverse samples to yield results that can be more readily generalized. Another potential weakness involves mathematical content bias. Since only the students' recent math study was used to compile the test, the results do not reflect mindfulness' effect on their participation in high-stakes tests on other mathematical content.

## Data availability statement

The raw data supporting the conclusions of this article will be made available by the authors, without undue reservation.

## Ethics statement

The studies involving human participants were reviewed and approved by Ethics Committee of Yangzhou University. Written informed consent to participate in this study was provided by the participants' legal guardian/next of kin.

## Author contributions

HZ contributed to the study conceptualization and design, produced the mindfulness audio, and conducted the data collection. HZ and LW collaborated in preparing the initial draft of the manuscript. LW edited the final manuscript. All authors contributed to the article and approved the submitted version.
